# A Novel Pyroptotic and Inflammatory Gene Signature Predicts the Prognosis of Cutaneous Melanoma and the Effect of Anticancer Therapies

**DOI:** 10.3389/fmed.2022.841568

**Published:** 2022-04-15

**Authors:** Yujian Xu, Youbai Chen, Zehao Niu, Jiahua Xing, Zheng Yang, Xiangye Yin, Lingli Guo, Qixu Zhang, Haixia Qiu, Yan Han

**Affiliations:** ^1^Department of Plastic and Reconstructive Surgery, The First Medical Center of Chinese PLA General Hospital, Beijing, China; ^2^Department of Plastic Surgery, The University of Texas MD Anderson Cancer Center, Houston, TX, United States; ^3^Department of Laser Medicine, The First Medical Center of Chinese PLA General Hospital, Beijing, China

**Keywords:** cutaneous melanoma, inflammatory response, immune infiltration, immune checkpoint, prognosis, pyroptosis, tumor microenvironment

## Abstract

**Purpose:**

The purpose of this study was to construct a gene signature comprising genes related to both inflammation and pyroptosis (GRIPs) to predict the prognosis of patients with cutaneous melanoma patients and the efficacy of immunotherapy, chemotherapy, and targeted therapy in these patients.

**Methods:**

Gene expression profiles were collected from The Cancer Genome Atlas. Weighted gene co-expression network analysis was performed to identify GRIPs. Univariable Cox regression and Lasso regression further selected key prognostic genes. Multivariable Cox regression was used to construct a risk score, which stratified patients into high- and low-risk groups. Areas under the ROC curves (AUCs) were calculated, and Kaplan-Meier analyses were performed for the two groups, following validation in an external cohort from Gene Expression Omnibus (GEO). A nomogram including the GRIP signature and clinicopathological characteristics was developed for clinical use. Gene set enrichment analysis illustrated differentially enriched pathways. Differences in the tumor microenvironment (TME) between the two groups were assessed. The efficacies of immune checkpoint inhibitors (ICIs), chemotherapeutic agents, and targeted agents were predicted for both groups. Immunohistochemical analyses of the GRIPs between the normal and CM tissues were performed using the Human Protein Atlas data. The qRT-PCR experiments validated the expression of genes in CM cell lines, Hacat, and PIG1 cell lines.

**Results:**

A total of 185 GRIPs were identified. A novel gene signature comprising eight GRIPs (TLR1, CCL8, EMP3, IFNGR2, CCL25, IL15, RTP4, and NLRP6) was constructed. The signature had AUCs of 0.714 and 0.659 for predicting 3-year overall survival (OS) in the TCGA entire and GEO validation cohorts, respectively. Kaplan-Meier analyses revealed that the high-risk group had a poorer prognosis. Multivariable Cox regression showed that the GRIP signature was an independent predictor of OS with higher accuracy than traditional clinicopathological features. The nomogram showed good accuracy and reliability in predicting 3-year OS (AUC = 0.810). GSEA and TME analyses showed that the high-risk group had lower levels of pyroptosis, inflammation, and immune response, such as lower levels of CD8+ T-cell infiltration, CD4+ memory-activated T-cell infiltration, and ICI. In addition, low-risk patients whose disease expressed *PD-1* or *CTLA-4* were likely to respond better to ICIs, and several chemotherapeutic and targeted agents. Immunohistochemical analysis confirmed the distinct expression of five out of the eight GRIPs between normal and CM tissues.

**Conclusion:**

Our novel 8-GRIP signature can accurately predict the prognosis of patients with CM and the efficacies of multiple anticancer therapies. These GRIPs might be potential prognostic biomarkers and therapeutic targets for CM.

## Introduction

Cutaneous melanoma (CM), the most aggressive skin cancer, accounts for <5% of skin cancers but more than 75% of skin cancer-related deaths (1). The incidence of CM increases at an average rate of 1.4% per year, with 87,000 new cases in the United States in 2017 alone (2). The 5-year survival rate of patients with early-stage CM is 93%, but that of patients with highly aggressive CM is <50%, and that of patients with advanced CM is only 15–20% (3, 4). Current methods to identify these high-risk patients are based on their clinicopathological characteristics. Accurate prediction of prognosis for individual patients is infeasible owing to the molecular heterogeneity and distinct tumor microenvironment (TME). This may explain that some patients with CM respond well to immunotherapies, targeted therapies, and chemotherapies, whereas others have innate and acquired resistance to these treatments. Improving the survival rates of these patients requires novel prognostic models and new predictive markers that can be used to tailor their treatment better.

Pyroptosis is an inflammation-dependent type of programmed cell death (5). Pyroptosis causes cell swelling and lysis to release vesicles containing inflammatory cytokines, such as interleukin (IL)-1 family members and high-mobility group box protein 1, and activate an intense inflammatory response (6). These inflammatory molecules affect the TME and are associated with tumorigenesis, tumor invasion, and metastasis (7, 8). Therefore, the pyroptosis-related molecules and their upstream genes may be potential biomarkers for a more accurate prognosis prediction for cancer patients. Indeed, recent studies have demonstrated that pyroptosis-related genes and long non-coding RNAs are promising prognostic predictors for many cancers, including pancreatic ductal adenocarcinoma (9), ovarian cancer (10), gastric cancer (11), lung cancers (12–15), glioblastoma (16), breast cancer (17–20), colorectal cancer (21, 22), thyroid cancer (23), bladder cancer (24, 25), head and neck squamous cell carcinoma (26–28), renal clear cell carcinoma (29, 30), endometrial cancer (31), and CM (32–36). However, how the pyroptosis-triggered inflammatory response alters the TME to affect the prognosis of patients with CM remains unclear. Therefore, comprehensive analyses of the correlation between pyroptosis- and inflammation-related genes in CM are required to develop new predictive biomarkers and prognostic models for a more precise treatment.

In this study, we constructed a novel gene signature comprising genes related to pyroptosis and inflammatory (GRIPs) to predict the prognosis of patients with CM and their responses to immunotherapies, chemotherapies, and targeted agents.

## Materials and Methods

### Data Acquisition and Normalization

RNA-seq data and corresponding clinical data were obtained from The Cancer Genome Atlas (TCGA; https://tcga-data.ncinih.gov/tcga/). The expression values of all genes were downloaded in fragments per kilobase of transcript per million mapped reads format. The RNA-seq data of normal skin were downloaded from the Genotype-Tissue Expression (GTEx) database (https://gtexportal.org/home/). The transcriptome samples included 471 tumor samples and 1 normal sample. Among them, 458 clinical samples were included for prognosis analysis because 13 patients had missing survival time and survival state. The clinical characteristics of the 458 patients are displayed in Supplementary Table 2. The merged RNA expression profile in TCGA-melanoma and GTEx-skin, including 471 CM samples and 234 normal samples, was normalized. The batch effects between TCGA and GTEX data were removed using the R software package “limma.” TCGA and Gene Expression Omnibus (GEO) data were normalized using R software packages “limma” and “sva” to remove biases from different platforms. We identified 40 pyroptosis-related genes and 675 inflammatory response-related genes (Supplementary Table 2) using the Molecular Signatures Database (http://www.gsea-msigdb.org/gsea/msigdb/).

### Identification of GRIPs

#### Co-expression Network Between Pyroptosis and Inflammation Genes

We used Pearson correlation to identify inflammatory response-related genes that are highly correlated with pyroptosis-related genes (R^2^ > 0.4 and *p* < 0.001). We used Cytoscape software (version 3.8.0) to visualize co-expression networks of pyroptosis-related genes and inflammatory response-related genes (37).

#### Pyroptosis Score Calculation

We used principal component analysis (PCA) (38) and gene set variation analysis (GSVA) (39) scores to assess pyroptosis in CM samples. PCA scores were based on the expression of the 40 pyroptosis-related genes, and both principal components 1 and 2 were selected to act as pyroptosis scores (33, 40). We define the pyroptosis score = ∑*PC*1*i* +*PC*2*i*, *i* is the expression of pyroptosis-related genes. GSVA, a method of gene set enrichment analysis (GSEA), was used to estimate the variation of the pyroptosis pathway (REACTOME PYROPTOSIS.gmt) over single CM samples in an unsupervised manner. Both the PCA score and GSVA score were recognized as the pyroptosis score, which represented the pyroptosis status of each sample. Patients with CM were divided into groups with either high or low pyroptosis scores using a cutoff value identified using the method of best separation in the R package survminer. Such grouping was used to minimize the *p*-value of the survival curve. Kaplan-Meier curves were depicted to compare the survival probability between patients with high and low pyroptosis scores.

#### Weighted Gene Co-expression Network Analysis

The expression profiles of the 675 inflammatory response-related genes were analyzed using weighted gene co-expression network analysis (WGCNA) to select modules of genes that were highly associated with pyroptosis PCA and GSVA scores. Co-expression networks were constructed using the R package WGCNA (41). Among the soft threshold values, the β that showed the highest mean connectivity (β = 3) was chosen. The module eigengene (ME) was associated with pyroptosis-related genes, PCA pyroptosis score, and GSVA pyroptosis score. Modules with the highest correlation were selected, and the genes of these modules were identified as GRIPs. Finally, the co-expression network method and the WGCNA modules genes were intersected to screen the final GRIPs.

### Identification of Differentially Expressed GRIPs

To identify differentially expressed GRIPs (DEGs), we analyzed the gene transcription data of the TCGA and Genotype-Tissue Expression (GTEx) datasets using the R package limma (42) with a false discovery rate < 0.05 and log2FC > 1. Heatmaps were conducted by using the R package “pheatmap.” Analyses of the Gene Ontology (GO) database (which includes biological process, cellular component, and molecular function information) and Kyoto Encyclopedia of Genes and Genomes (KEGG) database were used to evaluate the pathways associated with DEGs.

### Construction and Validation of a Prognostic GRIP Signature

To obtain a prognostic GRIP signature, we first performed univariable Cox regression to determine the association between DEGs and overall survival (OS). DEGs found to be correlated with OS (*p* < 0.05) in the univariable Cox regression were then selected for Lasso regression to further identify the key GRIPs according to dynamic coefficient profiling and lambda. Finally, we performed multivariable Cox regression analysis, including these key Lasso genes, to construct an optimal risk score. We used a heatmap to evaluate the relevance of the risk score and clinicopathological features.

The risk score was calculated as follows: risk score = $\sum_{i=1}^{n}\text{β}$i * (expression of GRIPs), where *n* is the number of key GRIPs and β is the regression coefficient. The risk score for each CM patient was calculated, and patients were classified as either low-risk or high-risk using the median risk score as the cutoff. All TCGA samples were randomly divided into a training dataset and a testing dataset at a 1:1 ratio. The GEO dataset GSE65904 was used as an external validation cohort. The validity of the risk score was verified in the training, testing, and entire cohort, as well as the external cohort using receiver operating characteristic (ROC) curves, risk plots, and survival analysis.

### Independent Predictor Identification and Nomogram Construction

To determine whether the risk score was an independent predictor of OS, we performed univariable and multivariable Cox regression analyses, which included the risk score, patient age, patient sex, melanoma stage, and TNM stage (T: tumor size; N: lymph node involvement; M: metastasis), in the entire cohort. Hazard ratios (HRs) and 95% confidence intervals (CIs) for each predictor were calculated.

The R packages “rgplot,” “survival,” and “rms” were used to construct nomograms (43) predictive of the 1-, 3-, and 5-year OS rates of patients with CM. The calibration curves of the nomograms in predicting 1-, 3-, and 5-year OS rates were constructed using the R package rms. The areas under the ROC curves (AUCs) for 3-year OS were predicted with independent predictors, and the nomogram was calculated to evaluate the predictive values of the GRIP signature and the nomogram.

### Gene Set Enrichment Analysis

We performed GSEA to identify the biological processes and pathways that were significantly alerted between the high-risk and low-risk subgroups based on the KEGG gene set “c2.cp.kegg.v7.4.symbols.gmt,” the Hallmark gene set “h.all.v7.4.symbols.gmt,” and other inflammation- and pyroptosis-related gene set. The TCGA gene expression was used as a phenotype label (44). The normalized enrichment score (NES) was calculated for each gene set. A nominal *p*-value of < 0.05 and a NES of >1.5 were used to identify significantly enriched pathways.

### Tumor Microenvironment Analysis

We used the CIBERSORT (45, 46), ESTIMATE (47, 48), MCPcounter (49, 50), single-sample GSEA (51), and TIMER algorithms (52) to compare the cellular components and immune responses between the high- and low-risk groups. Heatmaps were used to detect the differences in the immune response under different algorithms. In addition, ssGSEA was used to assess immune cell infiltration and function. Immune-related survival analysis was performed based on ssGSEA.

### Efficacies of Immune Checkpoint Inhibitors

We systematically identified 36 immune checkpoint-related genes that might be correlated with immune checkpoint inhibitors' (ICIs) response from previous studies. To compare the effect of ICI between the high- and low-risk groups, we assessed the programmed death 1 (PD-1) and cytotoxic T-lymphocyte antigen 4 (CTLA-4) using data from The Cancer Immunome Atlas (https://tcia.at/). TIDE algorithm was used to calculate the TIDE and Dysfunction scores to assess responsiveness to immunotherapy and level of immune dysfunction (http://tide.dfci.harvard.edu/) (53).

### Sensitivity Analysis of Chemotherapeutic and Targeted Agents

We used the “pRRophetic” package in R to evaluate the half-maximal inhibitory concentration (IC50) of common chemotherapy and molecular drugs to estimate the predictive role of the GRIP signature for CM treatment, such as rapamycin, docetaxel, and imatinib (54, 55).

### Verification of the Protein Expression of GRIPs

The protein expression levels of the GRIPs in normal and tumor tissues were verified using semiquantitative analysis of the immunohistochemical data extracted from The Human Protein Atlas (https://www.proteinatlas.org/), a database that includes immunohistochemistry-based expression data for ~20 of the most common types of cancers (56).

### Cell Culture

Human melanoma cell line: A375 was purchased from American Type Culture Collection (ATCC, America); SK-MEL-28 cell line was purchased from National Infrastructure of Cell Line Resource. Human immortalized keratinocytes cell line (Hacat) and human skin melanocyte cell line (PIG1) were purchased from Shanghai Guandao Biological Engineering Company with STR certifications. All cells were cultured in Dulbecco's modified Eagle's medium (Gibco) + 10% fetal bovine serum 10% fetal bovine serum under a humidified atmosphere of 37°C and 5% CO_2_.

### Real-Time Polymerase Chain Reaction (RT-PCR)

The real-time quantitative PCR (qRT-PCR) was performed to determine the relative expression levels of the genes. Total RNA was extracted using TRIzol reagent from two melanoma cells (A375, SK-MEL-28) and two control cells (Hacat, PIG1). The concentration of RNA was determined by ultraviolet spectrophotometry. Then, RNAs were reverse transcribed into cDNAs using the PrimerScript RT Master Mix. Finally, SYBR (Takara, Japan) was used to evaluate the mRNA expression levels. The mRNA expression levels of TLR1, IFNGR2, CCL25, IL15, CCL8, NLRP6, EMP3, and RTP4 were normalized by glyceraldehyde-3-phosphate dehydrogenase (GAPDH) that was used as the internal reference. The primer sequences of GAPDH and the eight genes are listed in Supplementary Table 3.

### Statistical Analysis

All statistical analyses were performed using R software (version 4.0.2). Cox regression and Lasso regression analyses were used to assess the predictive value of the GRIP signature. The Kaplan-Meier method was used to assess the survival of CM patients. The AUC was calculated from the ROC curve to assess prediction accuracy. Normally and nonnormally distributed variables were analyzed using the unpaired Student's *t*-test and the Wilcoxon test. All statistical tests were two-sided with *p* ≤ 0.05 being statistically significant.

## Results

### Identification of GRIPs

**Figure 1:**
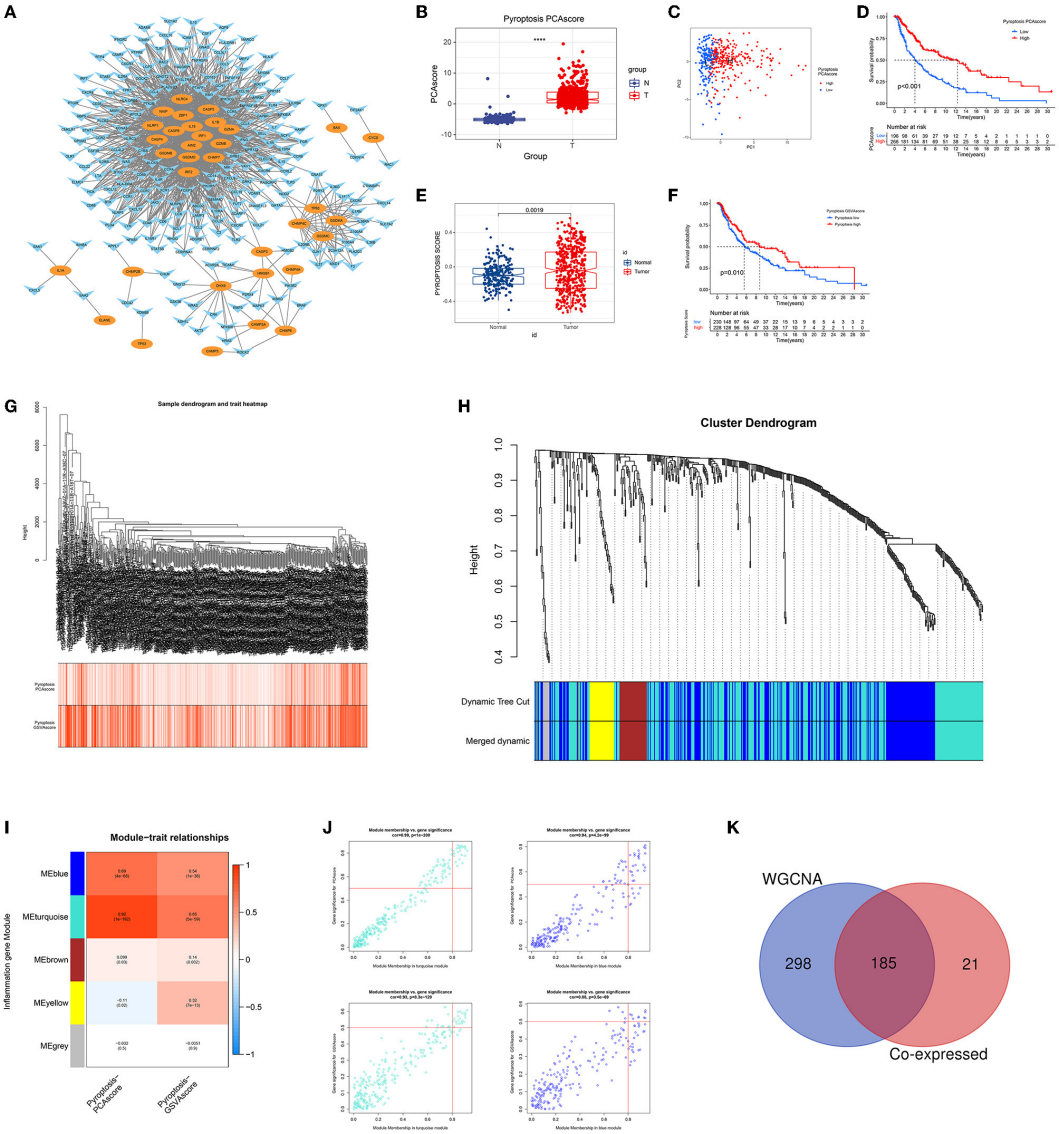
Identification of GRIPs. **(A)** The co-expression network of pyroptosis-related genes and inflammation-related genes. Oval: pyroptosis genes; horn: inflammatory genes. **(B)** CM had higher PCA pyroptosis scores than normal samples. **(C)** Visualization of high and low PCA pyroptosis scores. **(D)** Kaplan-Meier analysis showed that patients with high PCA pyroptosis scores had a higher survival probability. **(E)** CM had a higher GSVA pyroptosis score than normal samples. **(F)** Kaplan-Meier analysis showed that patients with high GSVA pyroptosis scores had a higher survival probability. **(G–J)** WGCNA identified pyroptotic inflammatory modules (ME blue and ME turquoise) based on the PCA and GSVA pyroptosis scores. **(K)** A Venn diagram illustrates that the co-expression network analysis and WGCNA have 185 overlapped genes, which are candidate GRIPs for constructing gene signature.

The co-expression network first identified 206 inflammatory response-related genes that were highly correlated with pyroptosis-related genes (Figure 1A). Using PCA pyroptosis scores, we found that the pyroptosis level of CM tissue was significantly higher than that of normal tissue (Figure 1B). Patients with CM were divided into high and low pyroptosis score groups (Figure 1C). The OS rate of patients with high pyroptosis scores was significantly higher than patients with low pyroptosis scores (Figure 1D). Similarly, GSVA showed that CM tissue had a higher pyroptosis level than normal tissue (Figure 1E) and patients with CM and high GSVA pyroptosis scores had higher OS rates than those with low GSVA pyroptosis scores (Figure 1F). WGCNA of the 675 inflammatory response-related genes identified 473 genes in the ME blue and ME turquoise modules that were highly related to PCA and GSVA pyroptosis scores (Figures 1G–J). The 206 genes obtained in the co-expression network and the 473 module genes obtained by WGCNA were intersected to identify 185 GRIPs (Figure 1K).

### Identifying the Differentially Expressed GRIPs

**Figure 2:**
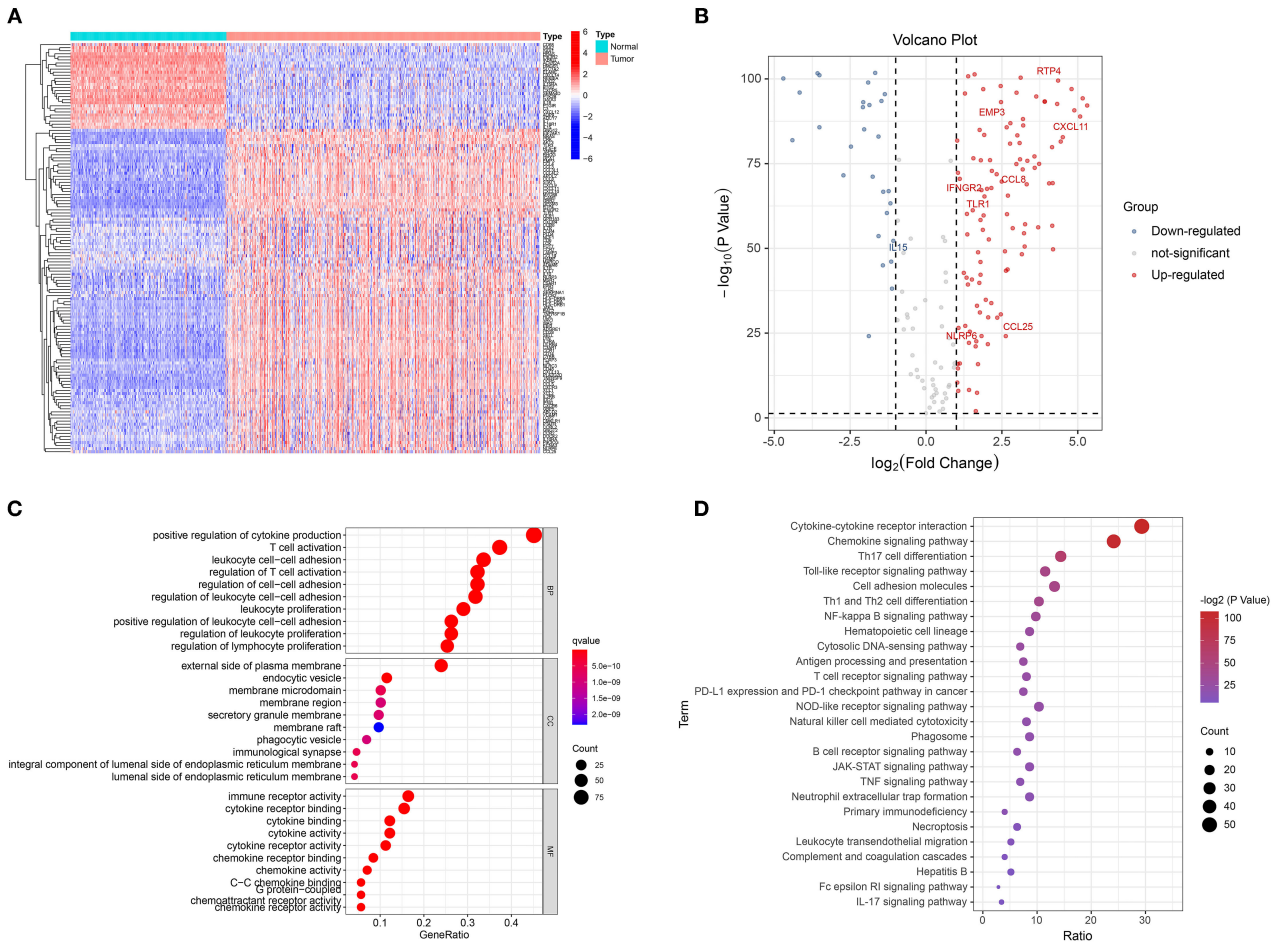
Identification of differentially expressed GRIPs. **(A)** Heatmap of differentially expressed GRIPs. **(B)** Volcano plot of differentially expressed GRIPs. **(C)** GO enrichment analysis of differentially expressed GRIPs. **(D)** KEGG enrichment analysis of differentially expressed GRIPs.

Of the 185 GRIPs, 134 were differentially expressed between tumor and normal tissue samples. In the tumor samples, 106 of these genes were upregulated compared with those in the normal tissue samples and 28 were downregulated (Figures 2A,B). GO analysis of the 134 DEGs showed that positive regulation of cytokine production, T-cell activation, leukocyte cell-cell adhesion, and regulation of T-cell activation was enriched in tumor samples (Figure 2C). KEGG analysis showed that cytokine-cytokine receptor interaction, chemokine signaling, Th17 cell differentiation, Toll-like receptor (TLR) signaling, PD-L1 expression, and PD-1 checkpoint pathways were significantly enriched in tumor samples (Figure 2D).

### Construction of a Prognostic GRIP Signature

**Figure 3:**
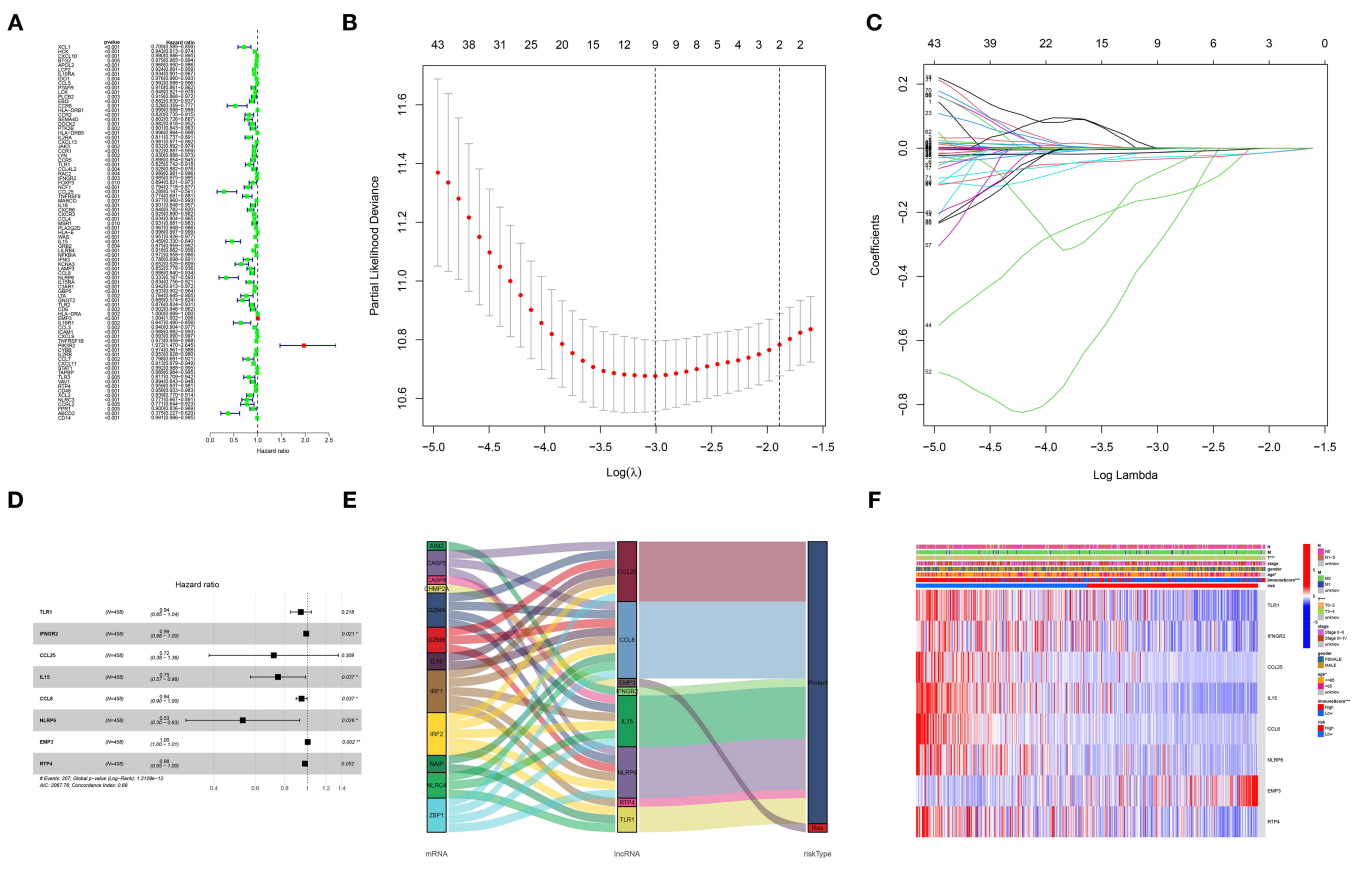
Construction of the GRIP signature. **(A)** Forest map of 83 prognostic GRIPs identified by univariable Cox regression. **(B,C)** Lasso regression lambda.min and lambda.1se criteria, parameter. **(D)** Multivariable Cox regression model. **(E)** A Sankey diagram shows the relationship between GRIPs and the risk type (risk or protect). **(F)** The heatmap illustrates the association between the eight GRIPs and clinicopathological characteristics.

Univariable Cox regression revealed that 83 of the 134 DEGs were correlated with OS (Figure 3A). Lasso regression identified nine prognostic GRIPs based on the optimal lambda value (Figures 3B,C). Then, the multivariate Cox regression finally identified eight GRIPs (EMP3, TLR1, IFNGR2, IL15, × CCL8, × NLRP6, CCL25, RTP4) for construction of the prognostic signature (Figure 3D). The risk score was calculated as Risk score = 0.003 × EMP3 exp-0.065 × TLR1 exp-0.012 × IFNGR2 exp-0.288 × IL15 exp-0.057 × CCL8 exp-0.633 × NLRP6 exp-0.329 × CCL25 exp-0.024 × RTP4 exp. The Sankey diagram showed the prognostic co-expression relationship between the pyroptosis genes and the GRIP signature (Figure 3E). A heatmap of the association between the risk signature and clinicopathological characteristics demonstrated that low immune scores were associated with higher risk scores (Figure 3F).

### Validation of the GRIP Signature

**Figure 4:**
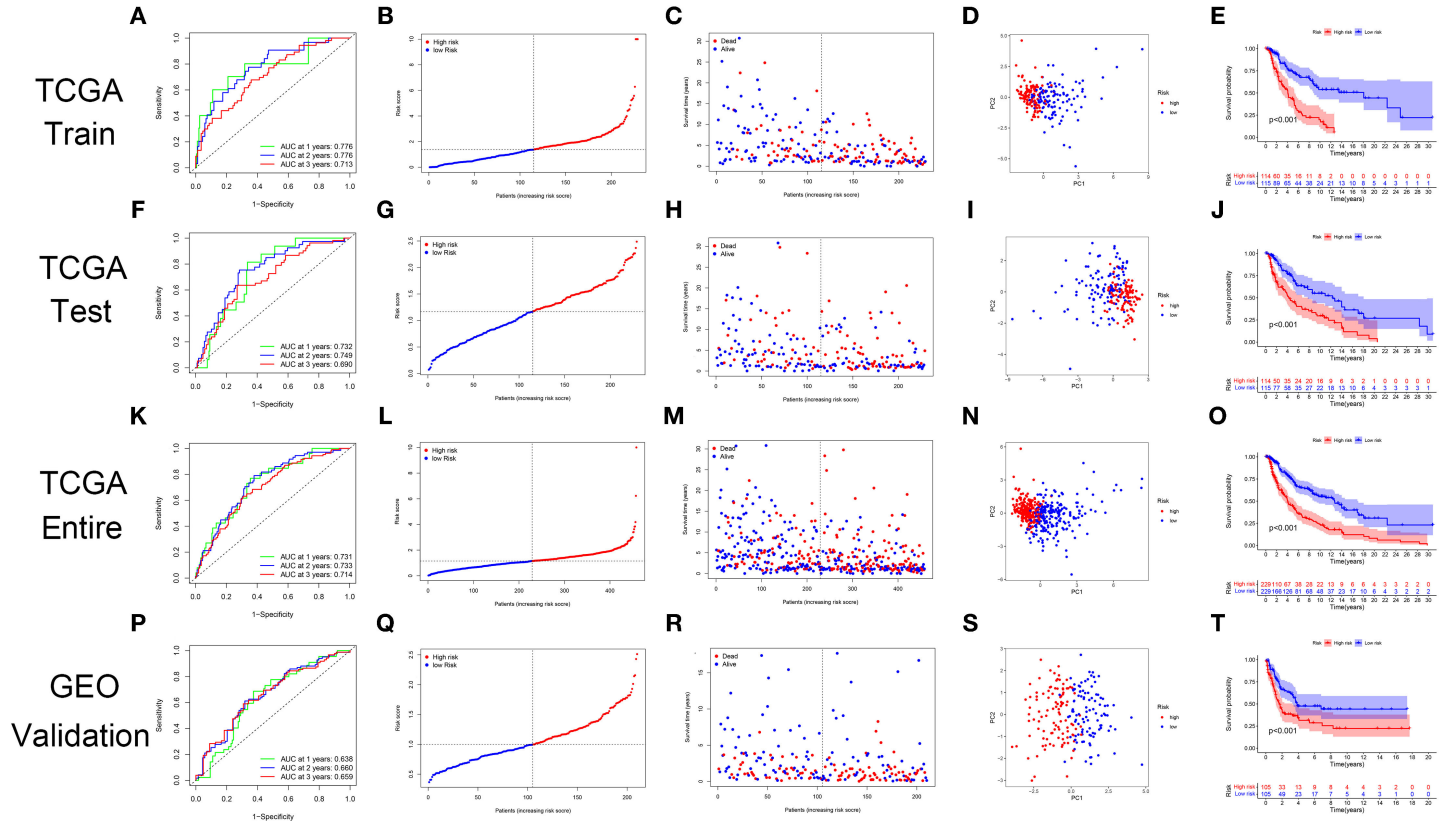
The GRIP signature has prognostic significance in patients with CM. **(A)** ROC curve in the TCGA training cohort. **(B,C)** The risk scores and statuses in the TCGA training cohort. **(D)** PCA of the TCGA training cohort. **(E)** Kaplan-Meier survival curves of the high- and low-risk groups in the TCGA training cohort. **(F–I)** The risk signature was verified in the TCGA testing cohort. **(J)** Kaplan-Meier survival curve of the patients in the TCGA testing cohort. **(K–O)** The risk signature was verified in the TCGA entire cohort. **(P–T)** The GEO dataset GSE65904 was used as an external validation dataset to verify the risk signature.

The GRIP signature had an AUC of 0.776, 0.776, and 0.713 for predicting 1-, 2-, 3-year OS in the training cohort (Figure 4A). The risk plots showed that the OS rates of patients gradually decreased as their risk scores gradually increased (Figures 4B,C). The patients in the training cohort were divided into high- and low-risk groups using the median risk score as a cutoff, and PCA showed the two groups to be well distinguished (Figure 4D). In the training cohort, the OS rate of the high-risk group was significantly lower than that of the low-risk group (Figure 4E). The prognostic GRIP signature was validated in the testing cohort and entire cohort (Figures 4F–O). External verification of the GRIP signature using the GEO dataset showed that the model had a fair ability to predict patient prognosis with an AUC of 0.638, 0.660, and 0.659 in predicting 1-, 2-, and 3-year ROC curve (Figures 4P–T).

### Independent Prognostic Value of the Risk Score and Nomogram

**Figure 5:**
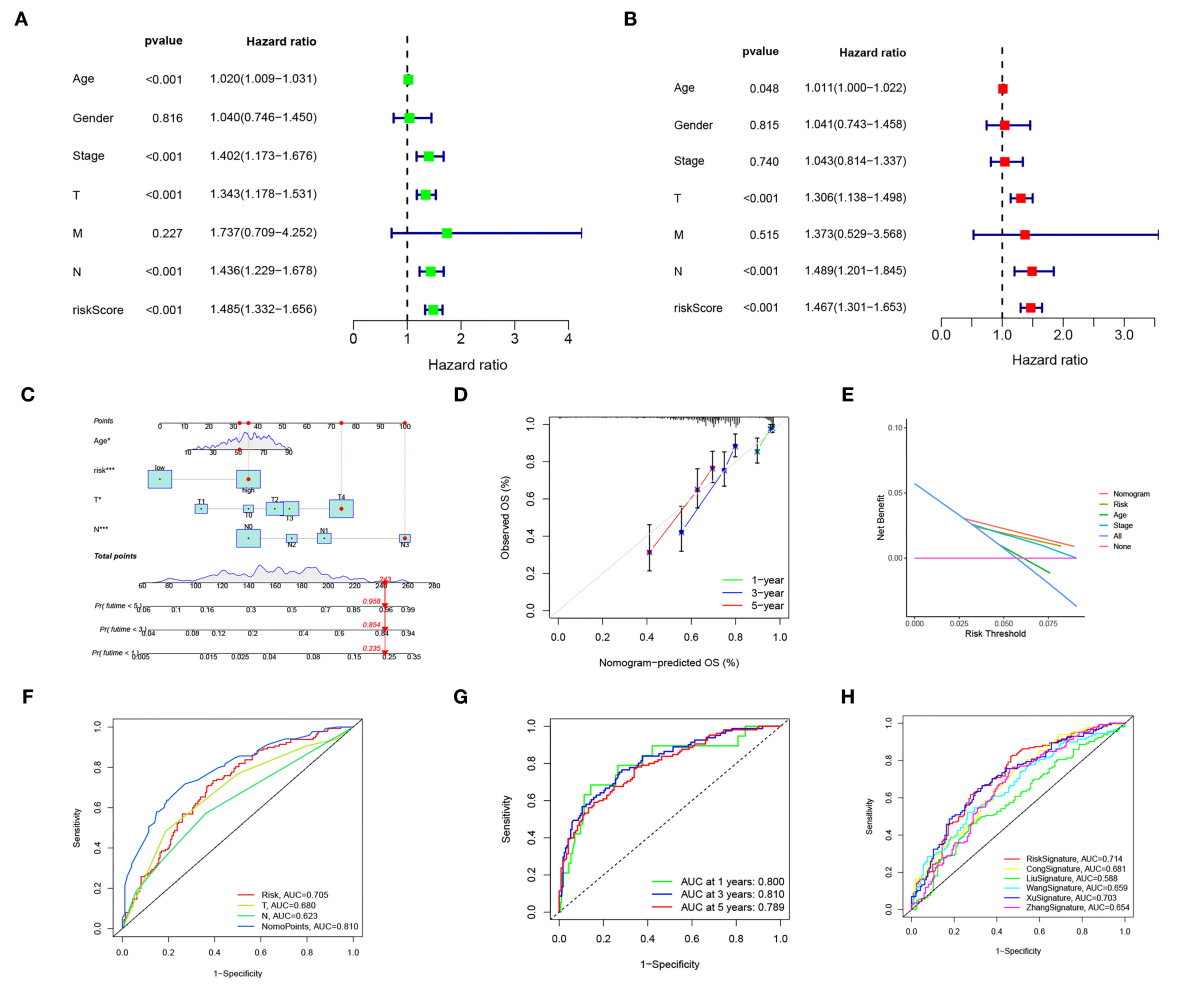
The risk score and nomogram have independent prognostic values. **(A)** Univariate Cox regression analyses of OS in the entire TCGA cohort. **(B)** Multivariate Cox regression analyses of OS in the entire TCGA cohort. **(C)** The nomogram, including clinical features and the risk score, for predicting outcomes in patients with CM. **(D)** The calibration curve analysis showed that the actual and the predicted 1-, 3-, 5-year survival times were consistent compared with the reference line. **(E)** A decision curve showed net benefits for patients with CM. **(F,G)** ROC curves for the nomopoints, risk scores, and clinical features. **(H)** ROC curves for the risk score and existing CM signature.

Univariable and multivariable Cox regressions showed that the risk score, age, T, and *n* were independent predictors for the OS of patients with CM (Figures 5A,B). The nomogram (Figure 5C) incorporating clinicopathological characteristics and the risk score was accurate in predicting the 1-, 3-, and 5-year OS rates of patients with CM (Figure 5D). A decision curve analysis proved that the signature and nomogram had a greater predictive ability than other traditional clinicopathological features for patients with CM net benefits (Figure 5E). The nomogram had an AUC of 0.810, showing a good predictive ability for the outcomes of patients with CM, which might be applied in the clinical management of patients with CM (Figures 5F,G). Compared with existing signatures for CM, the risk signature showed its performance better (Figure 5H).

### Gene Set Enrichment Analysis

**Figure 6:**
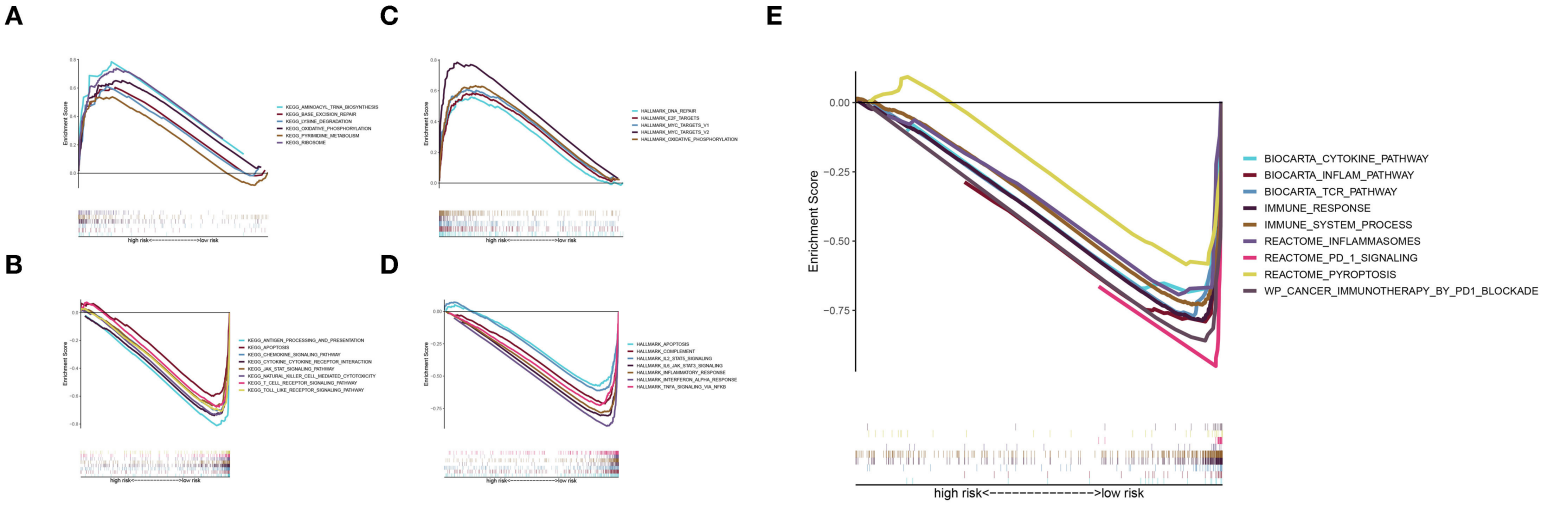
GSEA. **(A)** KEGG pathways of the signature in the high-risk group. **(B)** KEGG pathways of the signature in the low-risk group. **(C,D)** Hallmark pathway analysis revealed pathways enriched in the high-risk group **(C)** and low-risk group **(D)**. **(E)** Enrichment analysis of pyroptosis, immune-related, and inflammatory response-related pathways in the low-risk group.

The KEGG analysis indicated that RNA polymerase, aminoacyl tRNA biosynthesis, base excision repair, lysine degradation, pyrimidine metabolism, and oxidative phosphorylation were significantly activated in the high-risk group (Figure 6A), whereas the chemokine signaling pathway, apoptosis, JAK-STAT signaling pathway, natural killer cell, Toll-like receptor signaling pathway, cytokine-cytokine receptor interaction, T-cell receptor signaling pathway, and antigen processing and presentation were significantly activated in the low-risk group (Figure 6B). The Hallmark pathway analysis showed that DNA repair, E2F targets, MYC targets v1 and v2, and oxidative phosphorylation were upregulated in the high-risk group (Figure 6C), whereas IL2-STAT5 signaling, IL6-JAK-STAT3 signaling, inflammatory response, complement signaling, apoptosis, interferon α response, and TNFA signaling were upregulated in the low-risk group (Figure 6D). In addition, the inflammation pathway, *T*-cell receptor pathway, immune response, immune system process, inflammasomes, PD-1 signaling, and pyroptosis were upregulated in the low-risk group (Figure 6E). These results suggest that a pyroptosis-mediated inflammatory response upregulated the immune microenvironment and activated an inflammatory response in the low-risk group.

### Tumor Microenvironment Analysis

**Figure 7:**
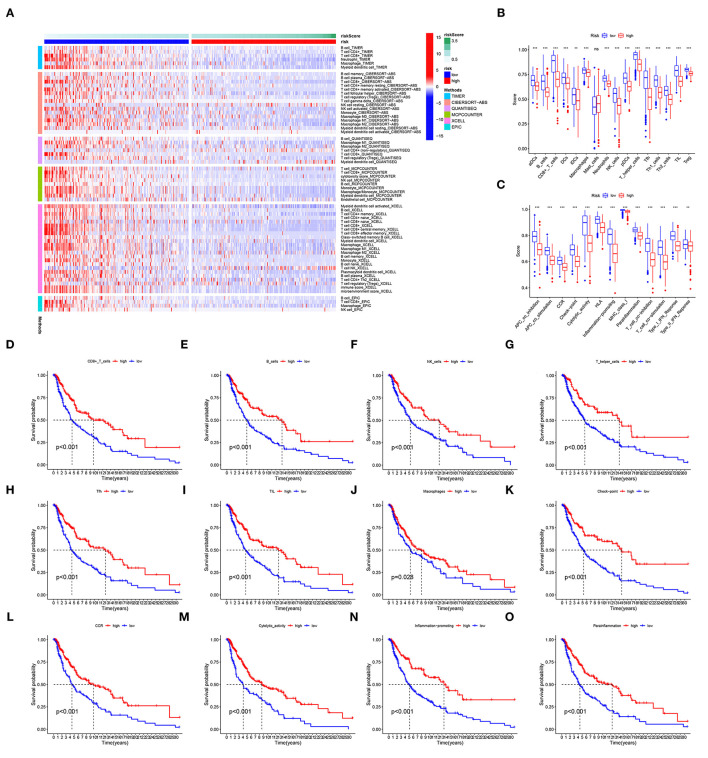
TME. **(A)** Immunocyte infiltration according to analyses with the CIBERSORT, ESTIMATE, MCPcounter, EPIC, XCELL, and TIMER algorithms. **(B,C)** The immune cells and functions identified with ssGSEAs. **(D–O)** The survival of patients is grouped by levels of immune checkpoints, CD8+ T cells, inflammation, TILs, and T helper cells. ns, not significant; **p < 0.01; ***p < 0.001.

The analysis of immunocyte infiltration indicated that immune cell subpopulations were highly enriched in the low-risk group compared to the high-risk group (Figure 7A). The ssGSEA of immune cells and functions showed that, compared with the high-risk group, the low-risk group had higher levels of immune checkpoints, cytolysis, human leukocyte antigens, inflammation, *T*-cell co-stimulation, T-cell co-inhibition, CD8+ T cells, T helper cells, and tumor-infiltrating lymphocytes (TILs) (Figures 7B,C). Differences in these factors, especially immune checkpoints, CD8+ T cells, B cells, inflammation, TILs, NK cells, T helper cells, and immune cells functions, had important impacts on the survival of patients (Figures 7D–O).

### Immune Checkpoints

**Figure 8:**
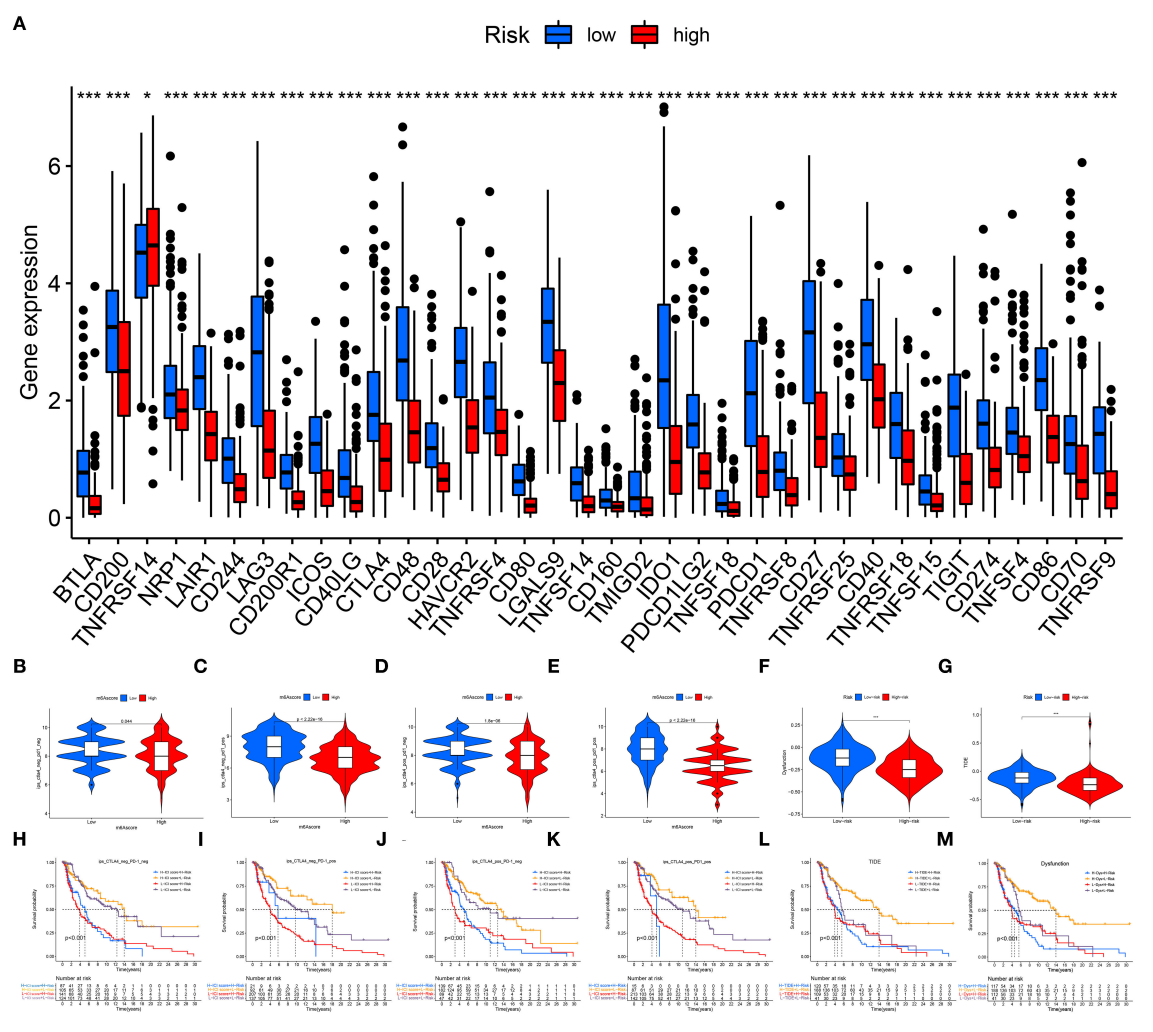
Immune checkpoint blockade. **(A)** A total of 36 immune checkpoints, including PD-L1, PD-1, and CTLA-4, were differentially expressed between high- and low-risk patients. **(B–E)** Immunotherapeutic effect of anti-PD-1 and anti-CTLA-4 antibodies in high- and low-risk patients. **(F)** TIDE evaluation. **(G)** Dysfunction evaluation. **(H–M)** The prognostic performance of immunotherapy cohorts. ns, not significant; *p < 0.05; ***p < 0.001.

A total of 36 immune checkpoints, including PD-L1, PD-1, and CTLA-4, were found to be differentially expressed between the low- and high-risk groups (Figure 8A). Compared with high-risk patients, low-risk patients had better responses to anti-PD-1 and anti-CTLA-4 immunotherapy (Figures 8B–E). The TIDE and dysfunction analyses confirmed that low-risk patients were more sensitive to immunotherapy (Figures 8F,G). The prognostic performance in immunotherapy cohorts showed that the patients with CM and a low-risk and high immunotherapy score had significant elevations in prognosis (Figures 8H–M).

### Drug Sensitivity Analysis

**Figure 9:**
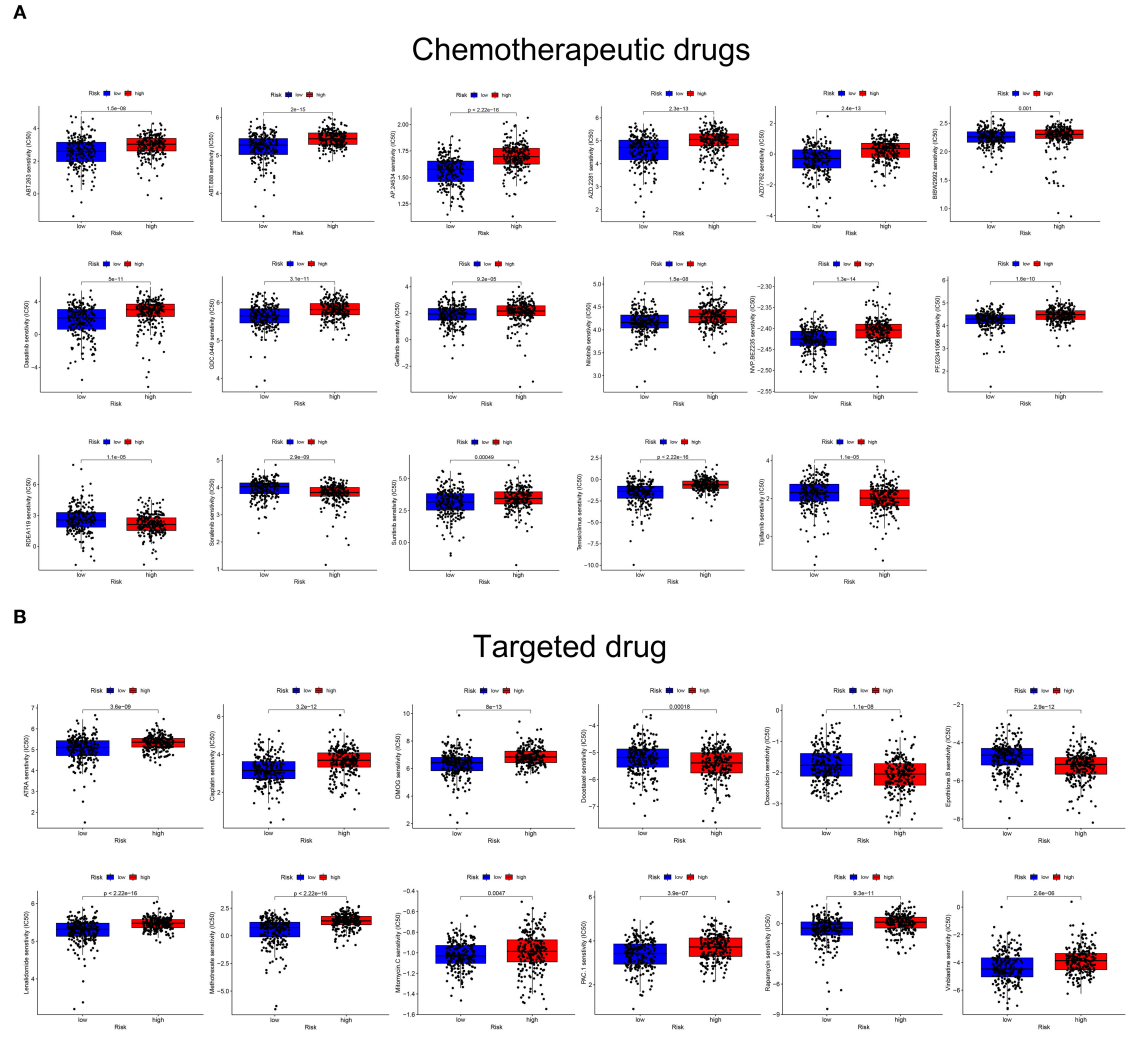
The GRIP signature is associated with chemotherapy and targeted therapy sensitivity. **(A)** The risk score was related to higher IC50 values of targeted agents. **(B)** The risk score was related to higher IC50 values of chemotherapeutic agents.

The estimated IC50 values for 17 common targeted drugs, such as afatinib, sorafenib, and refametinib, differed significantly between the two risk groups (Figure 9A). In addition, the low- and high-risk groups had significantly different sensitivity to 12 common chemotherapeutic drugs, such as docetaxel, rapamycin, cisplatin, and DMOG, and differed significantly between the two risk groups (Figure 9B). These results suggest that the GRIP signature can regulate sensitivity to chemotherapeutic and targeted drugs and that the risk model might be used to identify the potential biomarkers for chemotherapy and targeted therapy sensitivity.

### Verification of the MRNA and Protein Expression of Genes

**Figure 10:**
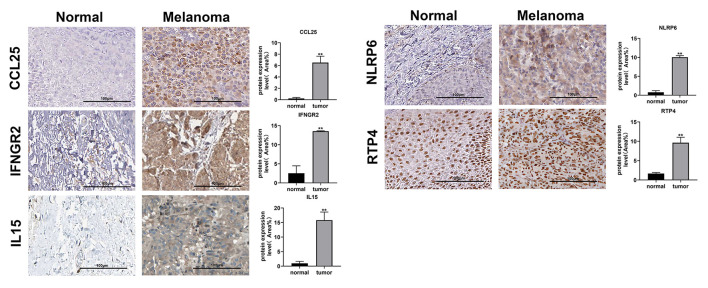
Verification of the protein expression of genes in the GRIP signature. Images from the Human Protein Atlas show the protein expression of IFNGR2, CCL25, IL15, NLRP6, and RTP4 in CM tissues and normal tissues. **p < 0.01.

**Figure 11:**
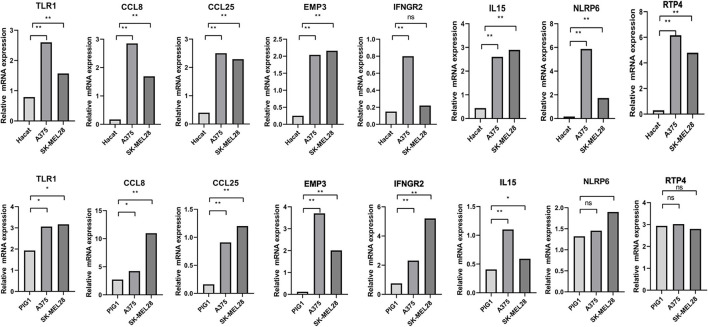
Validation of the expression of GRIPs in qRT-PCR. ns, not significant; *p < 0.05; **p < 0.01.

Immunohistochemical images of IFNGR2, CCL25, IL15, RTP4, and NLRP6 were obtained from The Human Protein Atlas database, and semiquantitative analysis confirmed the expression of these proteins in CM tissues (Figure 10). Then, the expression levels of the eight genes were validated in CM cell lines by qRT-PCR (Figure 11). The results showed that the expressions of TLR1, CCL8, EMP3, IFNGR2, CCL25, and IL15 were upregulated in A375 and SK-MEL-28 cell lines compared with Hacat and PIG1 cell lines. Although the expression of RTP4 and NLRP6 showed no statistical difference between PIG1 cells, A375 and SK-MEL-28 cell lines, the two genes were upregulated in A375 and SK-MEL-28 cell lines compared with Hacat cell.

## Discussion

### Major Findings

Our findings demonstrate that pyroptosis has antitumor activity and improves patient survival. Recent studies have shown that the antitumor effects of pyroptosis mainly depend on a strong inflammatory response and that pyroptosis changes the surrounding TME by triggering the release of inflammatory mediators (57, 58). Thus, we constructed a co-expression network and performed WGCNA to identify GRIPs and investigate their role in CM. Overall, our analyses identified 134 differentially expressed GRIPs. Our GO and KEGG analyses further showed that differentially expressed GRIPs were enriched in T-cell activation, cytokine and chemokine activity, and inflammatory response-related signaling pathways, such as the cytokine-cytokine receptor interaction, TLR, TNF, JAK-STAT, nuclear factor (NF)-κB, and NOD-like receptor (NLR) signaling pathways. After univariable Cox and Lasso regression analysis, we also constructed a GRIP risk signature, which we verified in the TCGA and GEO cohorts, and a nomogram scoring system for predicting the prognosis of patients with CM. Next, In addition, our GSEA showed that pyroptosis and inflammatory response have potential antitumor effects in patients with CM. Furthermore, our results also provide evidence of a complicated relationship among CM, the pyroptosis-mediated inflammatory response, and immunocyte infiltration in the TME. Drug sensitivity analysis indicated that GRIP signature might be used to identify potential biomarkers for chemotherapy and targeted therapy sensitivity. The immunohistochemical images of IFNGR2, CCL25, IL15, RTP4, and NLRP6 in both normal skin tissue and melanoma tissue confirmed the expression of the GRIPs in CM. Finally, the qRT-PCR experiments validated the expressions of our risk genes in Hacat, PIG1, A375, and SK-MEL-28 cell lines.

### GRIP Signature and Nomogram

Many kinds of pyroptosis or inflammation signatures have shown value in CM (32–36); however, there is almost no record about the genes both related to inflammation and pyroptosis models in CM prognosis and anticancer therapies. Our signature comprising eight GRIPs (TLR1, IFNGR2, CCL25, IL15, CCL8, NLRP6, EMP3, and RTP4) was superior to Lin‘s inflammation signature, an inflammatory response-related signature predicted the 3-year OS of patients with hepatocellular carcinoma with an AUC of 0.705 (59) and other researches' pyroptosis signature (10, 35) at 0.662 to 0.70. Our nomogram scoring system with an ROC curve of AUCs as 0.810 was higher than a recent study about signature nomogram in CM (60–65). In addition, all eight GRIPs we identified directly or indirectly play vital regulatory roles in molecular mechanisms. For example, Hu et al. have confirmed that the expression of TLR4 was related to prognosis and positively correlated with the infiltration of B cells, CD4 and CD8 T cells, neutrophils, macrophages, and dendritic cells in CM (66). Goto et al. showed that TLR2, TLR3, and TLR4 are highly expressed in human melanoma cells and that downstream signal transduction factors, such as NF-κB, and inflammatory response-related factors are activated in these cells (67). In another study, Sun et al. showed that NLR signaling pathways also play an important role in the inflammatory activity of the TLR4/NF-κB pathway and are associated with pyroptosis and the expression of downstream proinflammatory cytokines such as caspase-1, IL-1β, and IL-18 (68). The TLR4 we identified in GRIP signature was similar to those reported previously in patients with CM. In addition, IL15, CCL25, and CCL8 are associated with the inflammasome, which is a multiprotein complex whose assembly and activation are responsible for the recruitment and activation of caspases-1 and−5 that play an essential role in pyroptosis (68–70). EMP3 belongs to the peripheral myelin protein 22-kDa (PMP22) gene family as novel therapeutic targets in human cancer, which is the most important indicator of progression-free and metastasis-free survival for patients with urothelial carcinoma of the upper urinary tract (71). Therefore, our results suggested that the eight GRIPs might regulate pyroptosis by producing inflammation response. The functional enrichments we identified in CM were similar to those reported previously.

### Genes Related to Both Inflammation and Pyroptosis Signature in TME and Anticancer Therapies

In the TME, pyroptosis and the inflammatory response may enhance immune surveillance and antitumor immunity by recruiting TILs such as CD4+ and CD8+ T cells (72–74), and high levels of immune cell infiltration are associated with favorable outcomes (75). Our results further provide evidence of a complicated relationship among CM, the pyroptosis-mediated inflammatory response, and immunocyte infiltration in the TME. For instance, our signature mainly had a significantly positive correlation with the infiltration of neutrophils, CD8+ T cells, CD4+ T cells, and dendritic cells, which also had high expression levels of pyroptosis-related genes. Some studies have indicated that the level of immune infiltration in the TME (particularly that of immune cell subpopulations such as CD8+ T cells) is a crucial factor in the assessment of melanoma prognosis (76). Ahmadzadeh et al. reported that CD8+ T cells stimulate granulocytes and “produce perforin” or “produce granulocyte colony-stimulating factor and perforin” to kill tumor cells and that melanoma with high CD8+ T cell infiltration is more likely to respond to anti-PD-1/PD-L1 therapy (77). Similarly, we found that the GRIP signature was also highly correlated with PD-1 and CTLA-4 expression and with enhanced ICI efficacy and chemotherapeutic and targeted drug sensitivity in patients with CM. Thus, regulating the expression of GRIPs may increase the efficacy of ICB in melanoma, and our results also revealed the potential value of the GRIP signature to enhance the effect of immunotherapeutic, chemotherapeutic, and targeted drugs.

## Conclusion

The 8-GRIP prognostic signature we identified may be an independent prognostic factor for patients with CM. Our analyses of functional enrichments, the TME, ICI, and drug sensitivity verified that pyroptosis and inflammatory response play crucial roles in predicting the prognosis and immunotherapy response of CM. Finally, the qRT-PCR validated the expression of the eight genes in tumor cells. These findings may provide potential therapeutic targets in CM.

### Limitation

Although this study presents encouraging results, there are still several limitations in our study. First, the model was constructed and validated in open data source (TCGA and GEO). It would be better if its prognostic value was tested in another independent patient cohort. Second, our study lacked validation of clinical samples. Future studies are required to investigate the underlying mechanisms of GRIPs in mediating CM progression and immune microenvironment. We will continue to work on this in further studies.

## Data Availability Statement

The datasets presented in this study can be found in online repositories. The names of the repository/repositories and accession number(s) can be found in the article/Supplementary Material.

## Author Contributions

HQ and YH contributed to conception and design of the study. YX and YC were responsible for writing the whole article. ZN, JX, ZY, and XY performed the statistical analysis. ZN contributed to the subsequent basic experiments. GLL contributed to the idea of comprehensive evaluation. QZ contributed to instruct the technological process of the study. All authors contributed to the article and approved the submitted version.

## Conflict of Interest

The authors declare that the research was conducted in the absence of any commercial or financial relationships that could be construed as a potential conflict of interest.

## Publisher's Note

All claims expressed in this article are solely those of the authors and do not necessarily represent those of their affiliated organizations, or those of the publisher, the editors and the reviewers. Any product that may be evaluated in this article, or claim that may be made by its manufacturer, is not guaranteed or endorsed by the publisher.

## References

[1] Siegel R Miller K Jemal A. Cancer statistics, 2019. Ca Cancer J Clin. (2019) 69:7–34. 10.3322/caac.21551 30620402

[2] Trager M Queen D Samie F Carvajal R Bickers D Geskin L. Advances in prevention and surveillance of cutaneous malignancies. Am J Med. (2020) 133:417–23. 10.1016/j.amjmed.2019.10.008 31712100 PMC7709483

[3] Bsirini C Smoller B. Histologic mimics of malignant melanoma. Singapore Med J. (2018) 59:602–7. 10.11622/smedj.2018041 29774360 PMC6250758

[4] Zaenker P Lo J Pearce R Cantwell P Cowell L Lee M. A diagnostic autoantibody signature for primary cutaneous melanoma. Oncotarget. (2018) 9:30539–51. 10.18632/oncotarget.25669 30093967 PMC6078131

[5] Tsuchiya K. Switching from apoptosis to pyroptosis: gasdermin-elicited inflammation and antitumor immunity, *Int J Mol Sci*. (2021) 22:426. 10.3390/ijms22010426 33406603 PMC7794676

[6] Kovacs S Miao E. Gasdermins: effectors of pyroptosis. Trends Cell Biol. (2017) 27:673–84. 10.1016/j.tcb.2017.05.005 28619472 PMC5565696

[7] Broz P Pelegrín P Shao F. The gasdermins, a protein family executing cell death and inflammation. Nat Rev Immunol. (2020) 20:143–57. 10.1038/s41577-019-0228-2 31690840

[8] Zheng Z Li G. Mechanisms and therapeutic regulation of pyroptosis in inflammatory diseases and cancer. Int J Mol Sci. (2020) 21:456. 10.3390/ijms21041456 32093389 PMC7073143

[9] Tang R Liu X Liang C Hua J Xu J Wang W. Deciphering the prognostic implications of the components and signatures in the immune microenvironment of pancreatic ductal adenocarcinoma. Front Immunol. (2021) 12:648917. 10.3389/fimmu.2021.648917 33777046 PMC7987951

[10] Ye Y Dai Q Qi H. A novel defined pyroptosis-related gene signature for predicting the prognosis of ovarian cancer. Cell Death Discov. (2021) 7:71. 10.1038/s41420-021-00451-x 33828074 PMC8026591

[11] Shao W Yang Z Fu Y Zheng L Liu F Chai L. The pyroptosis-related signature predicts prognosis and indicates immune microenvironment infiltration in gastric cancer. Front Cell Dev Biol. (2021) 9:676485. 10.3389/fcell.2021.676485 34179006 PMC8226259

[12] Dong Z Bian L Wang M Wang L Wang Y. Identification of a pyroptosis-related gene signature for prediction of overall survival in lung adenocarcinoma. J Oncol. (2021) 2021:6365459. 10.1155/2021/6365459 34630565 PMC8497135

[13] Li X He J. A novel pyroptosis-related gene signature for early-stage lung squamous cell carcinoma. Int J Gen Med. (2021) 14:6439–53. 10.2147/IJGM.S331975 34675612 PMC8502038

[14] Lin W Chen Y Wu B Chen Y Li Z. Identification of the pyroptosis-related prognostic gene signature and the associated regulation axis in lung adenocarcinoma. Cell Death Discov. (2021) 7:161. 10.1038/s41420-021-00557-2 34226539 PMC8257680

[15] Song J Sun Y Cao H Liu Z Xi L Dong C. A novel pyroptosis-related lncrna signature for prognostic prediction in patients with lung adenocarcinoma. Bioengineered. (2021) 12:5932–49. 10.1080/21655979.2021.1972078 34488540 PMC8806662

[16] Li Xy Zhang Ly Li Xy Yang Xt Su Lx. A pyroptosis-related gene signature for predicting survival in glioblastoma. Front Oncol. (2021) 11:697198. 10.3389/fonc.2021.697198 34485134 PMC8416108

[17] Lv W Tan Y Zhao C Wang Y Wu M Wu Y. Identification of pyroptosis-related lncrnas for constructing a prognostic model and their correlation with immune infiltration in breast cancer. J Cell Mol Med. (2021) 25:10403–17. 10.1111/jcmm.16969 34632690 PMC8581320

[18] Ping L Zhang K Ou X Qiu X Xiao X. A novel pyroptosis-associated long non-coding rna signature predicts prognosis and tumor immune microenvironment of patients with breast cancer. Front Cell Dev Biol. (2021) 9:727183. 10.3389/fcell.2021.727183 34616734 PMC8488148

[19] Wu J Zhu Y Luo M Li L. Comprehensive analysis of pyroptosis-related genes and tumor microenvironment infiltration characterization in breast cancer. Front Immunol. (2021) 12:748221. 10.3389/fimmu.2021.748221 34659246 PMC8515898

[20] Xu D Ji Z Qiang L. Molecular characteristics, clinical implication, and cancer immunity interactions of pyroptosis-related genes in breast cancer. Front Med (Lausanne). (2021) 8:702638. 10.3389/fmed.2021.702638 34589498 PMC8473741

[21] Chen S Zhu J Zhi X. A novel pyroptosis-associated long noncoding rna signature to predict the prognosis of patients with colorectal cancer. Int J Gen Med. (2021) 14:6111–23. 10.2147/IJGM.S328842 34611426 PMC8485925

[22] Wei R Li S Yu G Guan X Liu H Quan J. Deciphering the pyroptosis-related prognostic signature and immune cell infiltration characteristics of colon cancer. Front Genet. (2021) 12:755384. 10.3389/fgene.2021.755384 34712271 PMC8546261

[23] Wu P Shi J Sun W Zhang H. Identification and validation of a pyroptosis-related prognostic signature for thyroid cancer. Cancer Cell Int. (2021) 21:523. 10.1186/s12935-021-02231-0 34627252 PMC8502398

[24] Chen X Chen H Yao H Zhao K Zhang Y He D. Turning up the heat on non-immunoreactive tumors: pyroptosis influences the tumor immune microenvironment in bladder cancer. Oncogene. (2021) 40:6381–93. 10.1038/s41388-021-02024-9 34588621

[25] Fu J Wang Y. Identification of a novel pyroptosis-related gene signature for predicting prognosis in bladder cancer. Cancer Invest. (2021) 21:1–17. 10.1080/07357907.2021.1991944 34644219

[26] Qian X Tang J Chu Y Chen Z Chen L Shen C. A novel pyroptosis-related gene signature for prognostic prediction of head and neck squamous cell carcinoma. Int J Gen Med. (2021) 14:7669–79. 10.2147/IJGM.S337089 34764680 PMC8575318

[27] Shen Y Li X Wang D Zhang L Li X Xia T. Novel prognostic model established for patients with head and neck squamous cell carcinoma based on pyroptosis-related genes. Transl Oncol. (2021) 14:101233. 10.1016/j.tranon.2021.101233 34600420 PMC8487076

[28] Zhu W Ye Z Chen L Liang H Cai Q. A pyroptosis-related lncrna signature predicts prognosis and immune microenvironment in head and neck squamous cell carcinoma. Int Immunopharmacol. (2021) 101:108268. 10.1016/j.intimp.2021.108268 34688154

[29] Sun Z Jing C Guo X Zhang M Kong F Wang Z. Comprehensive analysis of the immune infiltrates of pyroptosis in kidney renal clear cell carcinoma. Front Oncol. (2021) 11:716854. 10.3389/fonc.2021.716854 34568046 PMC8459616

[30] Tang X Zhang A Feng Y Su Y Wang X Jiang F. A novel pyroptosis-related lncrnas signature for predicting the prognosis of kidney renal clear cell carcinoma and its associations with immunity. J Oncol. (2021) 2021:9997185. 10.1155/2021/9997185 34764994 PMC8577956

[31] Zhang X Yang Q. A pyroptosis-related gene panel in prognosis prediction and immune microenvironment of human endometrial cancer. Front Cell Dev Biol. (2021) 9:705828. 10.3389/fcell.2021.705828 34722500 PMC8551636

[32] Ju A Tang J Chen S Fu Y Luo Y. Pyroptosis-related gene signatures can robustly diagnose skin cutaneous melanoma and predict the prognosis. Front Oncol. (2021) 11:709077. 10.3389/fonc.2021.709077 34327145 PMC8313829

[33] Meng J Huang X Qiu Y Zheng X Huang J Wen Z. Pyroptosis-related gene mediated modification patterns and immune cell infiltration landscapes in cutaneous melanoma to aid immunotherapy. Aging (Albany Ny). (2021) 13:24379–401. 10.18632/aging.203687 34753832 PMC8610130

[34] Wu L Liu G He Yw Chen R Wu Zy. Identification of a pyroptosis-associated long non-coding rna signature for predicting the immune status and prognosis in skin cutaneous melanoma. Eur Rev Med Pharmacol Sci. (2021) 25:5597–609. 10.26355/eurrev_202109_26779 34604952

[35] Wu Z Chen L Jin C Xu J Zhang X Yao Y. A novel pyroptosis-associated gene signature for immune status and prognosis of cutaneous melanoma. Peerj. (2021) 9:E12304. 10.7717/peerj.12304 34721986 PMC8520690

[36] Xie J Li H Chen L Cao Y Hu Y Zhu Z. A novel pyroptosis-related lncrna signature for predicting the prognosis of skin cutaneous melanoma. Int J Gen Med. (2021) 14:6517–27. 10.2147/IJGM.S335396 34675619 PMC8518699

[37] Shannon P Markiel A Ozier O Baliga Ns Wang Jt Ramage D. Cytoscape: a software environment for integrated models of biomolecular interaction networks. Genome Res. (2003) 13:2498–504. 10.1101/gr.1239303 14597658 PMC403769

[38] Fontes M Soneson C. The projection score–an evaluation criterion for variable subset selection in pca visualization. Bmc Bioinformatics. (2011) 12:307. 10.1186/1471-2105-12-307 21798031 PMC3167802

[39] Hänzelmann S Castelo R Guinney J. Gsva: gene set variation analysis for microarray and rna-seq data. Bmc Bioinformatics. (2013) 14:7. 10.1186/1471-2105-14-7 23323831 PMC3618321

[40] Zhang B Wu Q Li B Wang D Wang L Zhou Yl. M(6)a regulator-mediated methylation modification patterns and tumor microenvironment infiltration characterization in gastric cancer. Mol Cancer. (2020) 19:53. 10.1186/s12943-020-01170-0 32164750 PMC7066851

[41] Zhang B Horvath S. A general framework for weighted gene co-expression network analysis. Stat Appl Genet Mol Biol. (2005) 4:17. 10.2202/1544-6115.1128 16646834

[42] Ritchie Me Phipson B Wu D Hu Y Law Cw Shi W. Limma powers differential expression analyses for rna-sequencing and microarray studies. Nucleic Acids Res. (2015) 43:E47. 10.1093/nar/gkv007 25605792 PMC4402510

[43] Zhang Z Kattan M. Drawing nomograms with r: applications to categorical outcome and survival data. Ann Transl Med. (2017) 5:211. 10.21037/atm.2017.04.01 28603726 PMC5451623

[44] Subramanian A Tamayo P Mootha V Mukherjee S Ebert B Gillette M. Gene set enrichment analysis: a knowledge-based approach for interpreting genome-wide expression profiles. Proc Natl Acad Sci U S A. (2005) 102:15545–50. 10.1073/pnas.0506580102 16199517 PMC1239896

[45] Chen B Khodadoust M Liu C Newman A Alizadeh A. Profiling tumor infiltrating immune cells with cibersort. Methods Mol Biol. (2018) 1711:243–59. 10.1007/978-1-4939-7493-1_12 29344893 PMC5895181

[46] Newman A Liu C Green M Gentles A Feng W Xu Y. Robust enumeration of cell subsets from tissue expression profiles. Nat Methods. (2015) 12:453–7. 10.1038/nmeth.3337 25822800 PMC4739640

[47] Finotello F Trajanoski Z. Quantifying tumor-infiltrating immune cells from transcriptomics data. Cancer Immunol Immunother. (2018) 67:1031–40. 10.1007/s00262-018-2150-z 29541787 PMC6006237

[48] Yoshihara K Shahmoradgoli M Martínez E Vegesna R Kim H Torres-Garcia W. Inferring tumour purity and stromal and immune cell admixture from expression data. Nat Commun. (2013) 4:2612. 10.1038/ncomms3612 24113773 PMC3826632

[49] Shi J Jiang D Yang S Zhang X Wang J Liu Y. Lpar1, Correlated with immune infiltrates, is a potential prognostic biomarker in prostate cancer. Front Oncol. (2020) 10:846. 10.3389/fonc.2020.00846 32656075 PMC7325998

[50] Wang Z Wang Y Peng M Yi L. Ubash3b is a novel prognostic biomarker and correlated with immune infiltrates in prostate cancer. Front Oncol. (2019) 9:1517. 10.3389/fonc.2019.01517 32010618 PMC6974685

[51] Foroutan M Bhuva Dd Lyu R Horan K Cursons J Davis Mj. Single sample scoring of molecular phenotypes. Bmc Bioinformatics. (2018) 19:404. 10.1186/s12859-018-2435-4 30400809 PMC6219008

[52] Li T Fu J Zeng Z Cohen D Li J Chen Q. Timer2.0 for analysis of tumor-infiltrating immune cells. Nucleic Acids Res. (2020) 48:W509–509w514. 10.1093/nar/gkaa407 32442275 PMC7319575

[53] Jiang P Gu S Pan D Fu J Sahu A Hu X. Signatures of t cell dysfunction and exclusion predict cancer immunotherapy response. Nat Med. (2018) 24:1550-8. 10.1038/s41591-018-0136-1 30127393 PMC6487502

[54] Peng L Chen Y Ou Q Wang X Tang N. Lncrna miat correlates with immune infiltrates and drug reactions in hepatocellular carcinoma. Int Immunopharmacol. (2020) 89:107071. 10.1016/j.intimp.2020.107071 33221703

[55] Xu F Huang X Li Y Chen Y Lin L. Ma-Related lncrnas are potential biomarkers for predicting prognoses and immune responses in patients with luad. Mol Ther Nucleic Acids. (2021) 24:780-91. 10.1016/j.omtn.2021.04.003 33996259 PMC8094594

[56] Asplund A Edqvist Ph Schwenk Jm Pontén F. Antibodies for profiling the human proteome-the human protein atlas as a resource for cancer research. Proteomics. (2012) 12:2067-77. 10.1002/pmic.201100504 22623277

[57] Kesavardhana S Malireddi R Kanneganti Td. Caspases in cell death, inflammation, and pyroptosis. Annu Rev Immunol. (2020) 38:567–95. 10.1146/annurev-immunol-073119-095439 32017655 PMC7190443

[58] Man Sm Karki R Kanneganti T. Molecular mechanisms and functions of pyroptosis, inflammatory caspases and inflammasomes in infectious diseases. Immunol Rev. (2017) 277:61–75. 10.1111/imr.12534 28462526 PMC5416822

[59] Lin Z Xu Q Miao D Yu F. An inflammatory response-related gene signature can impact the immune status and predict the prognosis of hepatocellular carcinoma. Front Oncol. (2021) 11:644416. 10.3389/fonc.2021.644416 33828988 PMC8019928

[60] Hu B Wei Q Zhou C Ju M Wang L Chen L. Analysis of immune subtypes based on immunogenomic profiling identifies prognostic signature for cutaneous melanoma. Int Immunopharmacol. (2020) 89:107162. 10.1016/j.intimp.2020.107162 33168410

[61] Tian J Ma C Yang L Sun Y Zhang Y. Prognostic value and immunological characteristics of a novel rna binding protein signature in cutaneous melanoma. Front Genet. (2021) 12:723796. 10.3389/fgene.2021.723796 34531901 PMC8438157

[62] Tian M Yang J Han J He J Liao W. A novel immune checkpoint-related seven-gene signature for predicting prognosis and immunotherapy response in melanoma. Int Immunopharmacol. (2020) 87:106821. 10.1016/j.intimp.2020.106821 32731180

[63] Xie R Dong S Jiang J Yang C Li L Zhao S. Development and validation of an immune-related gene pair signature in skin cutaneous melanoma. Clin Cosmet Investig Dermatol. (2020) 13:973–86. 10.2147/CCID.S281364 33364806 PMC7751297

[64] Zeng F Su J Peng C Liao M Zhao S Guo Y. Prognostic implications of metabolism related gene signature in cutaneous melanoma. Front Oncol. (2020) 10:1710. 10.3389/fonc.2020.01710 33014847 PMC7509113

[65] Zhang Y Peng J Du H Zhang N Fang X. Identification and validation of immune- and stemness-related prognostic signature of melanoma. Front Cell Dev Biol. (2021) 9:755284. 10.3389/fcell.2021.755284 34805163 PMC8602573

[66] Hu J Xu J Feng X Li Y Hua F Xu G. Differential expression of the tlr4 gene in pan-cancer and its related mechanism. Front Cell Dev Biol. (2021) 9:700661. 10.3389/fcell.2021.700661 34631699 PMC8495169

[67] Goto Y Arigami T Kitago M Nguyen Sl Narita N Ferrone S. Activation of toll-like receptors 2, 3, and 4 on human melanoma cells induces inflammatory factors. Mol Cancer Ther. (2008) 7:3642–53. 10.1158/1535-7163.MCT-08-0582 19001446 PMC3480738

[68] Sun Z Nyanzu M Yang S Zhu X Wang K Ru J. Vx765 Attenuates pyroptosis and hmgb1/tlr4/nf-κb pathways to improve functional outcomes in tbi mice. Oxid Med Cell Longev. (2020) 2020:7879629. 10.1155/2020/7879629 32377306 PMC7181015

[69] Ferguson Sa Varma V Sloper D Panos Jj Sarkar S. Increased inflammation in ba21 brain tissue from african americans with Alzheimer's Disease. Metab Brain Dis. (2020) 35:121-33. 10.1007/s11011-019-00512-2 31823110

[70] Inoue M Williams Kl Gunn Md Shinohara Ml. Nlrp3 inflammasome induces chemotactic immune cell migration to the cns in experimental autoimmune encephalomyelitis. Proc Natl Acad Sci U S A. (2012) 109:10480-5. 10.1073/pnas.1201836109 22699511 PMC3387125

[71] Wang Yw Cheng Hl Ding Yr Chou Lh Chow Nh. Emp1, Emp 2, And emp3 as novel therapeutic targets in human cancer. Biochim Biophys Acta Rev Cancer. (2017) 1868:199–211. 10.1016/j.bbcan.2017.04.004 28408326

[72] Jefferies Ca. Regulating Irfs In Ifn driven disease. Front Immunol. (2019) 10:325. 10.3389/fimmu.2019.00325 30984161 PMC6449421

[73] Kumar S Calianese D Birge Rb. Efferocytosis of dying cells differentially modulates immunological outcomes in tumor microenvironment. Immunol Rev. (2017) 280:149–64. 10.1111/imr.12587 29027226 PMC8359679

[74] Ma Y Pitt Jm Li Q Yang H. The renaissance of anti-neoplastic immunity from tumor cell demise. Immunol Rev. (2017) 280:194–206. 10.1111/imr.12586 29027231

[75] Rivadeneira Db Depeaux K Wang Y Kulkarni A Tabib T Menk A. Oncolytic viruses engineered to enforce leptin expression reprogram tumor-infiltrating t cell metabolism and promote tumor clearance. Immunity. (2019) 51:548–60. 10.1016/j.immuni.2019.07.003 31471106 PMC6903394

[76] Marzagalli M Ebelt N Manuel E. Unraveling the crosstalk between melanoma and immune cells in the tumor microenvironment. Semin Cancer Biol. (2019) 59:236–50. 10.1016/j.semcancer.2019.08.002 31404607

[77] Ahmadzadeh M Johnson L Heemskerk B Wunderlich J Dudley M White D. Tumor antigen-specific cd8 t cells infiltrating the tumor express high levels of pd-1 and are functionally impaired. Blood. (2009) 114:1537–44. 10.1182/blood-2008-12-195792 19423728 PMC2927090

